# Apathy in patients with Neuromyelitis Optica Spectrum Disorder

**DOI:** 10.1371/journal.pone.0339479

**Published:** 2025-12-31

**Authors:** Zihan He, Wenjing Li, Xinyue Huang, Shuai Ma, Yuanbin Zhao, Shiquan Wang, Lili Yang

**Affiliations:** 1 Department of Neurology, Sichuan Academy of Medical Sciences & Sichuan Provincial People’s Hospital, University of Electronic Science and Technology of China, Chengdu, China; 2 Department of Neurology, Tongjiang County People’s Hospital, Bazhong, China; Universita degli Studi di Napoli Federico II, ITALY

## Abstract

**Background:**

Apathy is a common neuropsychiatric complication of neurological diseases, but it has not been investigated in patients with neuromyelitis optica spectrum disorders (NMOSD) until now.

**Methods:**

We enrolled 66 patients diagnosed with NMOSD and assessed apathy using the self-reported version of the Apathy Evaluation Scale (AES-S). All the patients also completed the investigations composed of demographic data, disease characteristics, and composite evaluations of life status, including anxiety/depression, fatigue, sleep, and quality of life. Further statistical analysis proceeded.

**Results:**

The mean AES-S score was 36.7 ± 8.3, with 40.9% of patients exhibiting clinically significant apathy (cutoff score: 36). Correlation analysis revealed that higher AES-S scores were significantly correlated with lower education attainment (p = 0.002), more number of attacks (p = 0.008), longer disease duration (p = 0.004), higher disability (p = 0.03), severer anxiety (p < 0.001), severer depression (p < 0.001), severer fatigue (p < 0.001), and severer sleep disturbances (p = 0.001). Depression was revealed to be a significant independent factor of apathy (P < 0.001). The subscales of AES-S and its correlated factors were also analyzed. Further analysis showed that the AES-S score was negatively correlated with the total score of the quality of life scale and all the sub-dimensions' scores (P < 0.05).

**Conclusions:**

These results suggest that apathy is a common neuropsychiatric complication in patients with NMOSD and is closely related to their quality of life. The apathy of NMOSD correlated with various physiological and psychological changes, especially depression. These findings might help us identify patients with a high risk of apathy, highlighting the importance of evaluating and managing apathy is of great significance for improving the quality of life in NMOSD patients.

## Introduction

Neuromyelitis optica spectrum disorders (NMOSD) is an autoimmune demyelinating disease of the central nervous system (CNS), with an estimated global pooled prevalence of 1.82 per 100,000 people [1]. The prevalence has been reported to be higher among Asian populations [2], whereas in the Chinese population, the prevalence is as high as 3.31/100,000 [3]. This disorder is characterized by recurrent inflammatory lesions in the optic nerve, spinal cord, brainstem, and cerebrum, thereby leading to severe motor and sensory impairments, bladder dysfunction, vision loss, and other debilitating symptoms [1,4–6]. The recovery of NMOSD patients is variable, and inflammatory attacks often result in permanent disability, even after the occurrence of a single episode [6–8].

As a chronic CNS disorder with unpredictable attacks, high disability and recurrence rates, NMOSD patients typically suffer unique psychological burden, including anxiety, depression, fatigue, cognitive dysfunction, and sleep disorders [9–12]. A recent meta-analysis of 31 studies revealed comorbidity rates of 40% for depression and 45% for anxiety in NMOSD patients [13]. These neuropsychiatric symptoms have been shown to significantly impair patients' quality of life [14–16]. Thus, it is highly important to comprehensively understand and manage these neuropsychiatric complications in patients with NMOSD.

Apathy is primarily described as a loss of motivation in early literature [17,18], but has been redefined by the presence of quantitative reduction of goal-directed activity either in the behavioral, cognitive, emotional, or social dimension in comparison to the patient's previous level of functioning in the recent 2018 international consensus [19]. It is a pervasive neuropsychiatric complication of neurological diseases, including multiple sclerosis (MS) [20–26], Alzheimer's disease (AD) [27], Parkinson's disease (PD) [28], vascular dementia [29], stroke [30], and cerebral small vessel disease (CSVD) [31]. It has been observed to be associated with poor treatment compliance, cognitive deficits, disability, and high caregiver distress in these neurological diseases [20,21,31–36]. Further, apathy was considered to be a predictor of progressive cognitive changes during MS [36]. Neuroimaging studies have revealed that the pathogenesis of apathy may involve damage to specific neural circuits; however, this damage varies across different diseases. For example, microstructural damage to the frontostriatal circuit has been suggested to be the pathological basis for apathy in MS patients [35], whereas damage to the mesocortical pathway may be linked to apathy in CSVD patients [37]. Therefore, apathy may be an important behavioral marker of CNS injury, and identifying apathy early in disease progression is considered a clinical and research priority. However, apathy has not received considerable attention in NMOSD patients.

Therefore, for the first time, we designed a cross-sectional study to assess the degree of apathy in NMOSD patients by the self-reported version of the Apathy Evaluation Scale (AES-S). Furthermore, we aimed to identify the factors correlated with apathy and to assess the impacts of apathy on quality of life in patients with NMOSD. We believe that our study can initially reveal the prevalence rate and related factors of apathy in NMOSD patients, which will provide a foundation for further research on neural mechanisms and intervention strategies.

## Materials and methods

### Participants

This was a cross-sectional study. NMOSD patients who visited the Neurology Department of Sichuan Provincial People's Hospital from Sep 2022 to Apr 2024 were consecutively recruited. All of the patients satisfied the diagnostic criteria according to the 2015 International Panel for Neuromyelitis Optica Diagnosis (IPND) criteria [4] and were verified according to the latest diagnostic criteria, which were updated in 2023 [38]. The exclusion criteria were as follows: (1) had a history of drug or alcohol abuse or other major clinical or psychiatric conditions; (2) were unable to complete all of the questionnaires with the assistance of neurologists; (3) had a disease duration of less than 3 months; and (4) were receiving acute immunoregulatory treatment. This study was approved by the Ethics Committee of Sichuan Provincial People's Hospital. The recruited participants provided written informed consent before enrolling in the study.

Patients' demographic data and disease characteristics were collected. The recorded disease characteristics included the presence of antibodies associated with CNS demyelinating disease in the serum (including AQP4-Ab, anti-myelin oligodendrocyte glycoprotein antibody (MOG-Ab), and anti-glial fibrillary acidic protein antibody (GFAP-Ab)), the number of clinical attacks, clinical phenotype, disease duration, degree of disability, and current preventive therapy. The degree of disability was independently assessed by two neurologists according to the Expanded Disability Status Scale (EDSS) [39].

### Composite evaluation of living status in NMOSD patients

a) Apathy

All of the patients completed the AES-S to evaluate apathy [40], which consists of 18 items scored on a 4-point Likert scale. The total score ranges from 18–72, with higher scores indicating more severe apathy symptoms. A cutoff value of 36 was used to identify patients with clinically significant apathy, which has been validated to have good sensitivity (88%) and specificity (72%) in MS patients [20,21,35]. Furthermore, the 18 items of AES had been categorized into cognitive subscale (8 items, Sub-C), behavioral subscale (5 items, Sub-B), emotional subscale (2 items, Sub-E), and other subscale (3 items, Sub-O, including the understanding of one's condition and evaluating overall motivation/initiative) [40].

b) Anxiety and depression

The Hospital Anxiety and Depression Scale (HADS) was developed to identify cases of anxiety disorders and depression among patients in nonpsychiatric hospital clinics [41]. This scale is divided into an anxiety subscale (HADS-A) and a depression subscale (HADS-D), both of which contain seven intermingled items. Each item is rated on a 4-point scale ranging from 0 to 3, with higher scores indicating more severe symptoms of anxiety or depression. The total scores of the HADS-A and HADS-D both range from 0 to 21.

c) Fatigue

The Brief Fatigue Inventory (BFI) is a one-dimensional questionnaire containing nine items that is used to assess the severity and impact of fatigue [42]. Each item of the inventory involves a scale with 11 points (ranging from 0 to 10). The total score is the average of all of the question scores (ranging from 0 to 10), with higher scores indicating more severe fatigue.

d) Sleep

The Pittsburgh Sleep Quality Index (PSQI) is a self-reported scale that is used to assess sleep disorders occurring over the month prior to the study [43]. The global PSQI score ranges from 0 to 21, with higher scores indicating worse sleep quality.

e) Quality of life

The 36-item short-form health survey (SF-36) is used to evaluate participants' quality of life [44]. The SF-36 includes the following eight dimensions: general health perception (GH), physical functioning (PF), role limitations due to physical problems (RP), bodily pain (BP), energy/vitality (VT), social functioning (SF), role limitations due to emotional problems (RE), and mental health (MH). Additionally, it includes one question that encompasses changes in health status that occurred over the previous year, which is specifically known as the reported health transition score. Each domain can be scored separately, with scores ranging from 0 (worst health state) to 100 (best health state). The Chinese version of the SF-36 has demonstrated a Cronbach's α between 0.72 and 0.88, and the test-retest reliability ranges from 0.66 to 0.94 [45].

All of the scales that were used in the present study have been utilized in previous NMOSD studies [46–49], except for the AES-S. All of the Chinese versions of these scales have been previously validated [48,50–52]. A psychologist administered the HADS questionnaires; the other questionnaires were completed by the participants themselves. A neurologist will provide necessary assistance nearby, only when the participants encounter difficulties in reading and understanding the items.

### Statistical analysis

All of the statistical analysis were performed using the statistical software GraphPad Prism (version 9, San Diego, CA). For all of the quantitative data, appropriate statistical methods were chosen based on their normality, which was checked with the Kolmogorov-Smirnov test.

To analyze the factors related to apathy in NMOSD patients, an unpaired T test (for normally distributed variables) or Mann-Whitney (for non-normally distributed variables) was used to determine whether the AES-S score and subscale scores differed among groups according to gender. One-way ANOVA (for normally distributed variables) and Kruskal-Wallis tests (for non-normally distributed variables) were chosen to determine the difference of AES-S score and subscale scores among groups distributed by clinical phenotype, and current preventive therapy.

Pearson's correlation analysis (for normally distributed variables) or nonparametric Spearman's ranked correlation analysis (for non-normally distributed variable) was conducted to explore the relationships between the AES-S score/subscale scores and the independent variables, including age, education years, number of attacks, disease duration, EDSS score, HADS-A score, HADS-D score, BFI score, and PSQI score. Multiple linear regression was used to further assess the independent factors of the AES-S score/subscale scores (by verification of the normality of the residuals). Age, education years, number of attacks, disease duration, EDSS score, HADS-A score, HADS-D score, BFI score, and PSQI score were included as possible independent variables for the multiple linear regression model. Furthermore, nonparametric Spearman's ranked correlation analysis was used to explore the relationships between the AES-S score and the SF-36 score/subscores according to the normality test. P < 0.05 was considered to be statistically significant.

## Results

### Demographic and clinical characteristics

This study included a total of 66 NMOSD patients. The average duration of the disease was 4.7 years (range: 0.25–23); moreover, the average number of attacks was 3.9 (range: 1–21), and the EDSS score was 3.0 (range: 0–7.5). In NMOSD patients, the positive rate of AQP4 antibodies was determined to be 81.8%, and the clinical subtypes included optic neuritis (ON, 21.2%), myelitis (TM, 34.8%), and optic neuromyelitis (ON+TM, 43.9%). Seven patients in the ON+TM subgroup also experienced other clinical syndromes (3 patients experienced area postrema syndrome, whereas 4 patients experienced cerebral syndromes). In terms of current preventive therapy, 42 patients (63.6%) were using immunosuppressants, including mycophenolate mofetil (MMF) and azathioprine (AZA); 19 patients (28.7%) were receiving treatment with B cell depletion therapy; and 5 patients (7.5%) were receiving other treatments, including 1 patient receiving treatment with satolizumab (SAT), 3 patients receiving oral administrations of low-dose prednisolone, and one patient who did not receive any therapy.

### Evaluation of apathy and its correlated factors in NMOSD patients

The AES-S score of the NMOSD patients was 36.7 ± 8.3 (mean ± SD), whereas 40.9% (27/66) of the patients demonstrated clinically significant apathy (a cutoff value of 36 was used). As for the subscale scores of NMOSD patients, the Sub-C score was 16.6 ± 4.3, the Sub-B scores was 10.4 ± 2.9, the Sub-E score was 4.5 ± 1.6, and the Sub-O score was 5.9 ± 1.6.

**Fig 1:**
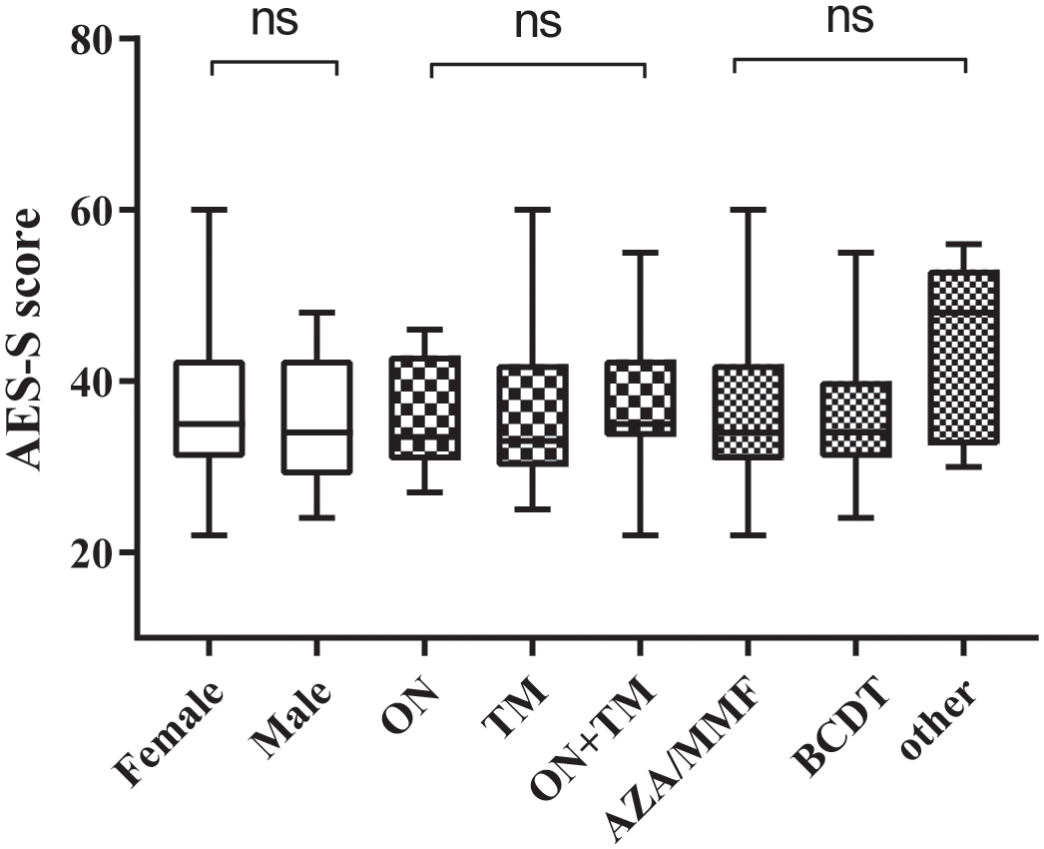
The comparisons of AES-S score among NMOSD subgroups distributed by gender, clinical phenotype, and current preventive therapy. AES-S, self-reported version of Apathy Evaluation Scale; ON, optica neuritis; TM, transverse myelitis; MMF, mycophenolate mofetil; AZA, azathioprine; BCDT, B cell depletion therapy; ns, not significant.

To investigate the factors correlated with apathy in NMOSD patients, we first analyzed the differences in AES-S scores among the NMOSD subgroups according to gender, clinical subtype, and current preventive therapy. We observed no significant differences in AES-S scores/subscale scores among NMOSD patients in terms of gender, clinical subtype, or therapy group (p > 0.05) (Fig 1). All statistical results are detailed in S1 Table.

**Fig 2:**
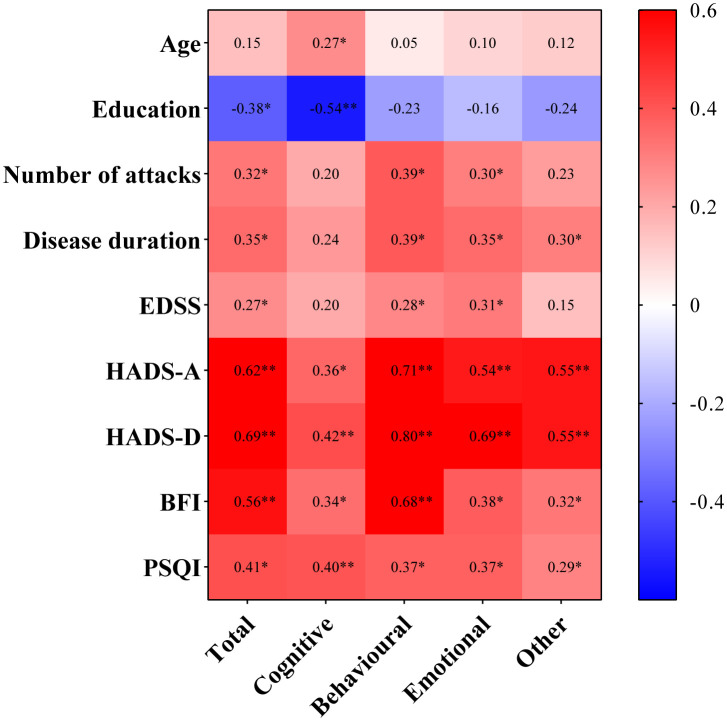
Heatmaps of the r values in the correlation analysis between the AES-S score/subscores and clinical variables in NMOSD patients. The r values are labeled. The correlations with statistical significance are marked with * (P < 0.05) or ** (P < 0.001). AES-S, self-reported version of Apathy Evaluation Scale; EDSS, Expanded Disability Status Scale; HADS, Hospital Anxiety and Depression Scale; BFI, Brief Fatigue Inventory; PSQI, Pittsburgh Sleep Quality Index.

In addition, we conducted separate correlation analyses between AES-S and subscale scores and other quantitative variables, such as age, years of education, and number of attacks, as well as EDSS, HADS-A, HADS-D, BFI, and PSQI scores (the results visualized as a heatmap in Fig 2). The results demonstrated that higher AES-S scores were significantly correlated with lower education attainment (r = −0.38, p = 0.002), a greater number of attacks (r = 0.32, p = 0.008), longer disease duration (r = 0.35, p = 0.004), higher EDSS scores (r = 0.27, p = 0.03), higher HADS-A scores (r = 0.62, p < 0.001), higher HADS-D scores (r = 0.69, p < 0.001), higher BFI scores (r = 0.56, p < 0.001), and higher PSQI scores (r = 0.41, p = 0.001) in NMOSD patients. The correlation between the AES-S subscale scores and clinical factors also exhibited distinct characteristics (Fig 2). Detailed statistical results are presented in S2 Table.

### Multivariate linear regression of the AES-S score in NMOSD patients

To investigate the independent factors of the AES-S score and subscale scores, we further conducted multiple linear regression analysis. The statistical results are displayed in Table 1. According to the multivariate linear regression analysis model, the AES-S score was only significantly and independently correlated with depression (HADS-D score, P < 0.001). As for the subscale scores of AES-S, we found that the Sub-E and Sub-B scores were both independently correlated with depression (HADS-D score, P < 0.001), while the Sub-B score also correlated with fatigue (BFI score, P < 0.001). Moreover, education year was found to be the only independently correlated factor of the Sub-C score (P = 0.01). No factors were found to independently correlate with the Sub-O score.

**Table 1:** Multivariable linear regression of the AES-S score/subscale scores in NMOSD patients.

Variables	B	S.E.	95%CI	*P*
**AES-S total score**				
Depression (HADS-D)	1.10	0.29	0.52 to 1.68	<0.001
**Cognitive subscale score (Sub-C)**				
Education year	0.31	0.05	0.20 to 0.41	<0.001
**Behavioural subscale score (Sub-B)**				
Depression (HADS-D)	0.36	0.08	0.20 to 0.53	<0.001
Fatigue (BFI)	0.38	0.11	0.15 to 0.60	0.002
**Emotional subscale score (Sub-E)**				
Depression (HADS-D)	0.31	0.052	0.20 to 0.41	<0.001

AES-S, self-reported version of Apathy Evaluation Scale; HADS-D, Hospital Anxiety and Depression Scale – depression; BFI, Brief Fatigue Inventory;

### The impact of apathy on quality of life in NMOSD patients

To assess the impact of apathy on the quality of life of NMOSD patients, we assessed the relationships between the AES-S score and the SF-36 scores using Spearman's rank correlation analysis. The results revealed that the AES-S score was negatively correlated with the total score of the SF-36 (r = −0.50, P < 0.001) and the scores of all of the subdimensions (r: −0.61–0.27, P < 0.05), except for the reported health transition score (r = −0.16, P = 0.19). The detailed statistical results are displayed in Table 2.

**Table 2:** The correlations between the AES-S score and the SF-36 score.

SF-36 subscore/total score	r	95% CI	P value
Physical Functioning	**−0.33**	**−0.56 to −0.09**	**0.007**
Role Physical	**−0.27**	**−0.49 to −0.02**	**0.03**
Bodily Pain	**−0.30**	**−0.51 to −0.06**	**0.01**
General Health	**−0.49**	**−0.66 to −0.28**	**<0.001**
Vitality	**−0.61**	**−0.75 to −0.43**	**<0.001**
Social Functioning	**−0.48**	**−0.65 to −0.27**	**<0.001**
Role Emotional	**−0.39**	**−0.58 to −0.16**	**0.001**
Mental Health	**−0.56**	**−0.71 to −0.36**	**<0.001**
Reported Health Transition	−0.16	−0.40 to 0.09	0.19
Total score	**−0.50**	**−0.67 to −0.29**	**<0.001**

AES-S, self-reported version of Apathy Evaluation Scale; SF-36, the 36-item short-form health survey.

## Discussion

Apathy is a common neuropsychiatric complication of neurological diseases; however, it has not been investigated in NMOSD patients until now. Our study revealed that 40.9% of NMOSD patients exhibited clinically significant apathy. Further analysis revealed that the degree of apathy was significantly negatively correlated with the level of education and positively correlated with disease duration, number of attacks, disability (by the EDSS score), anxiety (by the HADS-A score), depression (by the HADS-D score), fatigue (by the BFI score), and sleep disorders (by the PSQI score) in NMOSD patients. Multivariate analysis indicated that depression was the only independent factor of apathy. Notably, apathy was strongly negatively correlated with patients’ quality of life (by the SF-36 score), thereby suggesting that it could be a critical intervention target for improving the overall quality of life of NMOSD patients.

According to the updated diagnostic criteria from the 2018 International consensus group, apathy was defined as a quantitative reduction of goal-directed activity in comparison to the patient's previous level of functioning(Criterion A), with symptoms persisting for at least four weeks, and affecting at least two of the three apathy dimensions (behaviour/cognition; emotion; social interaction, Criterion B). Moreover, the symptoms of apathy should also fit the criteria of causing identifiable functional impairments (Criterion C) and should not be fully explained by other factors (Criterion D) [19]. Specifically designed assessment scales for apathy screening enable both clinicians and patients to promptly identify and evaluate the burden of apathy symptoms. The AES is a widely used tool that assesses participants' general condition over the past four weeks, evaluating multiple sub-dimensions of apathy, such as behavioral, cognitive, emotional, and social interaction [40]. Although first published in 1991, the questionnaire aligns with the detailed requirements of the Criteria A and B in 2018 diagnostic criteria. Further, the cutoff value of 36 used in our study (equivalent to an average score of 2 per item) also satisfies Criterion C's requirements for symptom severity. Moreover, the AES has demonstrated excellent reliability and validity (Cronbach's α = 0.87–0.90) and has been successfully applied to various neurological disorders, particularly MS [20,53,54]. Self-rated apathy had been demonstrated to be highly correlated with clinician-rated apathy in the development of AES [40]. Therefore, we assume the definition of apathy via AES-S largely meets the latest diagnostic criteria.

Based on this preliminary study, approximately four out of the ten NMOSD patients were observed to suffer from clinically significant apathy in this study, which was comparable to its prevalence in MS patients (20–50%) [20–26], as well as the prevalence in AD (49%) [27], PD (16.5–40%) [28], CVSD (37.5%) [31], and stroke (33.0%) [30], highlighting the fact that apathy is a common neuropsychiatric complication of neurological diseases.

To further investigate the mechanisms underlying apathy in patients with NMOSD, we conducted a correlation analysis between apathy and its potential contributing factors, including demographic features, clinical characteristics and life-status evaluations. Our findings revealed that in terms of demographic and clinical characteristics, apathy was significantly associated with lower educational attainment, longer disease duration, more attacks, and more severe disability (via EDSS scores) in NMOSD patients. These findings were consistent with those of previous studies in MS patients [20,21,35]. However, in terms of the correlation with EDSS scores, apathy in NMOSD patients was less strongly correlated with EDSS when compared to previous findings in MS (r = 0.27 in this study; r = 0.38–0.40 in MS studies) [20,21]. This discrepancy may be attributed to the fact that the disability of NMOSD patients is primarily influenced by optic neuritis and myelitis (rather than brain lesions). This lesion-related retrograde neuronal damage and the extensive white matter microstructural damage that may accumulate during the course of the disease may constitute the pathophysiological basis for apathy in NMOSD patients. These findings highlight the close relationship between apathy and disease severity, thereby suggesting that apathy could serve as an important marker of disease progression.

In addition, the study revealed that educational attainment was negatively correlated with the total AES-S score and independently negatively correlated with cognitive subscale scores, highlighting the relationship between low educational attainment and a higher risk of apathy. Similarly, MS research has found that high levels of education may offer protection against cognitive decline, as they may reflect the protective effect of high cognitive reserves on cognitive decline [55]. Thus, it can be concluded that NMOSD patients with lower educational attainment should be closely monitored for the occurrence of impaired cognition and apathy.

Through a detailed investigation of the patients' life status and further multiple linear regression analysis, we also revealed that there were strong associations between apathy and elevated levels of depression in NMOSD patients, which were consistent with the findings in MS [35]. As a chronic condition with potential for sudden relapse and severe disability, it has been established that many NMOSD patients experience significant emotional disorders [12,13]. This study suggests that patients with depression in NMOSD may be at higher risk of developing apathetic symptoms. The co-occurrence of apathy and depression was also revealed in PD [56], AD [57], MS [35], and Huntington's disease [58].

Since apathy and depression share overlapping symptoms such as loss of interest, lack of initiative and social withdrawal, it also makes it harder to identify people with depression who are also apathetic. It should be noted that they are considered to be distinct neuropsychiatric disorders [59,60]. In terms of the symptoms and clinical evaluations, the hallmark symptoms of apathy are diminished initiative, diminished interest, and diminished emotional expression/responsiveness as described by the diagnostic criteria [19,60]. Symptoms like sadness, hopelessness, guilt, tearfulness, and suicidal ideation are specific to depression and may not be present in those with apathy [61]. Previous study determined convergence and divergence in the constructs of apathy and depressive symptoms, and determined that depression was defined most highly by items assessing sadness, low self-esteem, and loneliness, while apathy was characterized by poor motivation, low interest, and lack of initiative via Exploratory Factor Analyses (EFAs) [62]. Furthermore, the clinician-rated and self-rated versions of the AES have been demonstrated to be able to discriminate apathy from depression [40,63]. The subclass analysis of this study revealed that depression primarily affected the scores of emotional and behavioral subscales of apathy, while cognitive and other subscales remained independent of emotional evaluation. This suggested that apathy and depression were interrelated yet independent complications in NMOSD, while understanding the differences in the presentation of symptoms is important for us to identify apathy, especially when combined with depressive symptoms.

Apart from the differences in symptoms, the distinction between apathy and depressive symptoms is supported by differential associations with neuropsychological domains and cognitive impairment. A recent four-year observational study of MS patients revealed that baseline apathy could predict cognitive decline, whereas baseline depressive symptoms demonstrated no such correlation [55], which was consistent with previous studies indicating that apathy and depression are distinct clinical constructs with specific neural underpinnings [22,59,64,65]. Also, in patients with PD, both apathy and depression were associated with poorer verbal memory, whereas only apathy was associated with poorer executive function [66]. Future research is needed to explore the interaction between apathy and depression in NMOSD patients, and to elucidate the underlying neural substrates of apathy.

Different from other chronic neurological disease, the unpredictable and disabling nature of NMOSD creates a unique psychological burden on patients. Previous researchers conducted comprehensive analyses of psychological burden in NMOSD patients and their caregivers/partners through self-reporting and in-depth interviews, proposing unique psychological intervention strategies [12,67]. Further, they suggested that a previously unknown aspect of the psychological burden in NMOSD patients was the cognitive fusion, which can be understood as rigid attachment to thoughts as truth [12]. For example, having thoughts like ‘I can't do anything because of NMOSD’ and letting these thoughts prevent oneself from trying new activities. This may also be a psychological mechanism of apathy in NMOSD patients. As recommended in previous studies [12,68], we also highly recommend that NMOSD patients undergo regular psychological assessments and seek mental health services promptly throughout the course of the disease.

There are also several limitations of this study. First, as a cross-sectional observational study with a limited sample size, this study failed to evaluate the impact of apathy on disease prognosis. Second, this study did not include a cognitive evaluation or neuroimaging analysis. Further studies with larger sample sizes and the utilization of more comprehensive investigations are warranted to gain more knowledge of the underlying pathogenesis and intervention targets of apathy in NMOSD patients. Moreover, the self-rated assessment scales cannot fully replace experienced neuropsychological experts in making definitive clinical diagnoses through detailed interviews, and the analysis and discussion in the neuropsychological field remains insufficiently thorough. Future research should involve experienced neuropsychological experts to definitively diagnose, thereby better elucidating the neural mechanisms underlying apathy. This will also help establish specific cutoffs for assessment tools like AES in NMOSD.

## Conclusions

In conclusion, the present study revealed that apathy is a common neuropsychiatric complication in patients with NMOSD and is closely related to their quality of life. The apathy of NMOSD patients was revealed to be influenced by a combination of various demographic characteristics, disease characteristics, and life status changes, especially depression. These findings may help in the identification of patients who are at high risk of apathy and suggest that the evaluation and management of apathy is highly important for improving the quality of life of NMOSD patients. Further studies are warranted to explore the underlying pathogenesis and to identify interventions for apathy in NMOSD patients.

## Supporting information

S1 Table The comparisons of AES-S total score and subcale scores among NMOSD subgroups distributed by gender, clinical phenotype, and current preventive therapy. (DOCX)

S2 Table The correlation between AES-S score/subscore and clinical variables in NMOSD patients. (DOCX)

S3 Table The raw data of the study. (XLSX)
