# Supplementary material for: Apathy in patients with Neuromyelitis Optica Spectrum Disorder
Source: PLoS One. 2025 Dec 31;20(12):e0339479. doi: 10.1371/journal.pone.0339479 (PMC12755817; doi:10.1371/journal.pone.0339479)
Supplement: S1 Table — (DOCX) [file pone.0339479.s001.docx]

**Supplementary Table S1. The comparisons of AES-S total score and subcale scores among NMOSD subgroups distributed by gender, clinical phenotype, and current preventive therapy.**

| NMOSD subgroup | AES-S total score, mean [SD] (range) | *P* | Cognitive subscale score, mean [SD] (range) | *P* | Behavioural subscale score, median [SD] (range) | *P* | Emotional subscale score, median [SD] (range) | *P* | Other subscale score, median [SD] (range) | *P* |
| --- | --- | --- | --- | --- | --- | --- | --- | --- | --- | --- |
| **Gender** |  | 0.58 |  | 0.61 | 0.2706 | -0.5406 | 0.2049 | 0.2368 |  | 0.21 |
| Female | 37.1 [8.5] (22-60) |  | 16.8 [4.3] (8-28) |  | 0.02326 to 0.4867 | -0.6959 to -0.3371 | -0.04633 to 0.4318 | -0.01281 to 0.4587 | 6.330 [1.5] (3-9) |  |
| Male | 35.2 [7.5] (24-48) |  | 15.9 [4.7] (9-22) |  | 9.8 [2.3] (7-14) |  | 4.6 [0.9] (3-6) |  | 5.5 [1.9] (3-9) |  |
| **Phenotype** |  | 0.37 |  | 0.45 |  | 0.33 |  | 0.08 |  | 0.64 |
| ON | 35.4 [6.2] (27-46) |  | 15.6 [4.3] (8-24) |  | 10.2 [2.3] (6-14) |  | 3.9 [0.9] (2-6) |  | 5.7 [1.5] (4-9) |  |
| TM | 36.5 [9.7] (25-60) |  | 17.4 [4.7] (11-28) |  | 9.8 [3.0] (5-17) |  | 4.3 [1.6] (2-8) |  | 5.9 [1.6] (4-9) |  |
| ON+TM | 37.5 [8.2] (22-55) |  | 16.4 [4.0] (9-24) |  | 11.0 [3.0] (5-17) |  | 5.0 [1.7] (2-8) |  | 6.1 [1.6] (3-9) |  |
| **Therapy** |  | 0.26 |  | 0.05 |  | 0.11 |  | 0.08 |  | 0.91 |
| MMF/AZA | 36.2 [8.4] (22-60) |  | 16.8 [4.3] (8-27) |  | 9.9 [3.0] (5-17) |  | 4.3 [1.5] (2-8) |  | 5.9 [1.6] (3-9) |  |
| BCDT | 35.8 [7.1] (24-55) |  | 15.1 [3.6] (12-24) |  | 11.1 [2.5] (5-17) |  | 4.5 [1.5] (2-8) |  | 6.1 [1.3] (4-9) |  |
| other | 43.8 [10.9] (30-56) |  | 20.2 [5.8] (14-28) |  | 11.8 [3.0] (7-14) |  | 6.0 [1.6] (4-8) |  | 6.0 [2.0] (4-9) |  |

AES-S, the self-reported version of the Apathy Evaluation Scale; ON, optica neuritis; TM, transverse myelitis; MMF, mycophenolate mofetil; AZA, azathioprine; BCDT, B cell depletion therapy.
