# Supplementary material for: Apathy in patients with Neuromyelitis Optica Spectrum Disorder
Source: PLoS One. 2025 Dec 31;20(12):e0339479. doi: 10.1371/journal.pone.0339479 (PMC12755817; doi:10.1371/journal.pone.0339479)
Supplement: S2 Table — (DOCX) [file pone.0339479.s002.docx]

**Supplementary Table S2. The correlation between** **AES-S score/subscore and clinical variables in NMOSD patients.**

| Variables | AES-S total score | | Cognitive subscale score | | Behavioural subscale score | | Emotional subscale score | | Other subscale score | |
| --- | --- | --- | --- | --- | --- | --- | --- | --- | --- | --- |
|  | r | *P* | r | *P* | r | *P* | r | *P* | r | *P* |
| Age, years | 0.15 | 0.23 | **0.27** | **0.03** | 0.05 | 0.70 | 0.10 | 0.44 | 0.12 | 0.33 |
| Education years | **-0.38** | **0.002** | **-0.54** | **<0.001** | -0.23 | 0.06 | -0.16 | 0.21 | -0.24 | 0.06 |
| Number of attacks | **0.32** | **0.008** | 0.20 | 0.10 | **0.39** | **0.001** | **0.30** | **0.02** | 0.23 | 0.06 |
| Disease duration, years | **0.35** | **0.004** | 0.24 | 0.06 | **0.39** | **0.001** | **0.35** | **0.004** | **0.30** | **0.02** |
| EDSS | **0.27** | **0.03** | 0.20 | 0.11 | **0.28** | **0.02** | **0.31** | **0.01** | 0.15 | 0.22 |
| Anxiety (HADS-A) | **0.62** | **<0.001** | **0.36** | **0.003** | **0.71** | **<0.001** | **0.54** | **<0.0001** | **0.55** | **<0.001** |
| Depression (HADS-D) | **0.69** | **<0.001** | **0.42** | **<0.001** | **0.80** | **<0.001** | **0.69** | **<0.0001** | **0.55** | **<0.001** |
| Fatigue (BFI) | **0.56** | **<0.001** | **0.34** | **0.01** | **0.68** | **<0.001** | **0.38** | **0.001** | **0.32** | **0.01** |
| Sleep quality (PSQI) | **0.41** | **0.001** | **0.40** | **<0.001** | **0.37** | **0.002** | **0.37** | **0.002** | **0.29** | **0.02** |

AES-S, the self-reported version of the Apathy Evaluation Scale; r, Spearman's ranked correlation; EDSS, Expanded Disability Status Scale; HADS, Hospital Anxiety and Depression Scale; PSQI, Pittsburgh Sleep Quality Index; BFI, Brief Fatigue Inventory.
